# Current and Emerging Bladder Cancer Urinary Biomarkers

**DOI:** 10.1100/tsw.2011.104

**Published:** 2011-05-26

**Authors:** Justin Parker, Philippe E. Spiess

**Affiliations:** Department of Genitourinary Oncology, H. Lee Moffitt Cancer Center, Tampa, FL, USA

**Keywords:** bladder cancer, urinary cytology, FISH, NMP22, BTA stat, Immunocyt, telomerase, hyaluronic acid, microsatellite analysis, BLCA-4

## Abstract

Bladder cancer continues to be one of the most common malignancies. Those who have been already diagnosed are at high risk for recurrence, especially if the pathology demonstrates high-grade disease. Diagnosis and surveillance is reliant on invasive evaluation with cystoscopy. Urinary cytology has been used to aid in diagnosis, but its use is limited. Other assays have been developed that may aid in clinical decision making. The ultimate goal will be the development of a highly sensitive and specific urinary marker for bladder cancer. This would provide a noninvasive means of diagnosing the disease and limit the number of unnecessary cystoscopies. This article will review the currently available urinary bladder cancer markers. It will also review new and investigational urinary markers that have shown promise for future clinical use.

## INTRODUCTION

In 2010, an estimated 70,530 new cases of bladder cancer were diagnosed, with 14,680 deaths resulting from the disease[1]. Bladder cancer ranks in the top five of newly diagnosed cancers in men and in the top ten of estimated cancer deaths, ranking only behind prostate cancer in the category of genitourinary malignancies. At 1 year post-transurethral resection, patients with low- and medium-risk disease will have up to 20 and 40% tumor recurrence, respectively. Those with high-risk disease demonstrate even higher rates of recurrence, with up to 90% at 1 to 2 years postresection[2]. As such, close and accurate disease surveillance is paramount to the treatment of this malignancy and the prevention of progression to invasive disease. Diagnosis of bladder cancer has long relied on cystoscopy. This has been aided with the use of urine cytology; however, low sensitivity, particularly for low-grade disease, is associated with this test and has resulted in significant limitations. The advent of noninvasive urine-based markers, including fluorescence *in situ* hybridization (FISH), nuclear matrix protein 22 (NMP22), bladder tumor antigen (BTA), Immunocyt, as well as other novel modalities, has yielded improved diagnostic accuracy. This review will summarize the currently available data in regards to current and emerging urinary bladder cancer markers.

## CYTOLOGY

Urinary cytology has been established as the standard noninvasive urinary marker for detecting bladder cancer. The usefulness of cytology is dependent on the shedding of cancer cells into the urine and their proper identification by a pathologist. High-grade cancers are more likely than low-grade tumors to shed their cells into the urine due to their weaker intercellular attachments[3]. As has been demonstrated in multiple studies, this results in a poor sensitivity for low-grade bladder cancer, with improved sensitivity and specificity for high-grade disease. The sensitivity for low-grade cancers has been demonstrated to range from 13 to 75% and over 80% for high-grade cancers. The overall sensitivity of cytology ranges from 25 to 70%[3,4,5,6]. Pooled estimates from 36 studies and involving 14,260 patients demonstrated a sensitivity of 44% (95% CI, 38–51%) and a specificity of 96% (95% CI, 94–98%)[7].

The accuracy of cytology is dependent on analysis by a pathologist and classification into one of four categories: normal, atypical/indeterminate, suspicious, or malignant. As a diagnostic modality, it has been noted to have high inter- and intraobserver variability with a flat learning curve requiring years of training[4]. Findings as well can be limited by variation in specimen processing. Despite these limitations, the high specificity with relatively low expense has resulted in cytology being established as the backbone of urine-based bladder cancer diagnostic markers.

Quantitative cytology (Quanticyt) is an automated karyometric image analysis that allows for the quantitative grading of urine cytology[8]. Each imaged cell nucleus is analyzed for DNA content and nuclear morphometry[9]. The samples are then subsequently divided into low, intermediate, and high risk for recurrence of bladder cancer. This technique is limited by the necessity of a bladder wash sample for analysis to provide a large enough quantity of cells for analysis. A review of five studies involving 129 patients demonstrated a sensitivity of 58% (range: 45–65%) and a specificity of 76% (range: 68–87%) across 227 patients[10]. Van der Poel et al. evaluated 129 patients and demonstrated a sensitivity of 69% and a specificity of 72.5%[8]. The sensitivity increased to 85% for grade 3 tumors. Similar results have also been reported in a separate study of 105 patients with an overall sensitivity of 42.1% and specificity of 67.9%[11].

## FLUORESCENCE *IN SITU* HYBRIDIZATION (FISH)

FISH is a molecular test that utilizes DNA probes to identify the most common urothelial carcinoma–related chromosomal changes in exfoliated cells in the urine. Urovysion® FISH is a multitarget, multicolor assay developed by Vysis-Abbott Laboratories. The test was designed to detect aneuploidy of chromosomes 3, 7, 17, and the loss of the 9p21 locus in malignant urothelial cells[12]. The initial description of the Urovysion FISH test identified a positive result as five or more cells with a gain of two or more chromosomes, 10 or more cells with a gain of at least one chromosome, or 10 or more cells with homozygous loss of the 9p21 locus[13]. However, cutoff criteria may vary between institutions. This initial study by Sokolova et al. demonstrated a sensitivity of 84%, with a specificity of 92%. Multiple follow-up studies have shown the sensitivity of the Urovysion FISH test to be between 69 and 85%, with a specificity of 78 to 92%[3,7,13,14,15]. In a direct comparison of FISH with cytology and NMP22, FISH was found to have better sensitivity for low-grade tumors. The combination of FISH and cytology detected 97.4% of cancers, while the combination of cytology and NMP22 detected 92.1% of tumors[16].

The apparent ability for FISH to detect up to half of recurrent bladder tumors prior to being visualized on cystoscopy has led to the description of an “anticipatory positive”[15]. In cases with positive FISH and negative cystoscopy, a significantly decreased time to recurrence over those with negative FISH and negative cystoscopy has been found. FISH has also been demonstrated to be of use in monitoring response to intravesical therapy with Bacillus Calmette Guerin (BCG). Patients with a positive post-therapy FISH have been shown to develop recurrence earlier and are 9.4 times as likely to have muscle-invasive disease as those with a negative post-therapy FISH[17].

## NUCLEAR MATRIX PROTEIN 22 (NMP22)

NMPs are an important part of the structural framework of the nucleus. These proteins have important roles in DNA replication, transcription, and regulation of gene expression. NMP22 is specific for the nuclear mitotic apparatus protein that is involved with the proper distribution of chromatids to daughter cells. It has been demonstrated that NMP22 concentration is 25 times greater in bladder cancer cell lines than in the urothelium from normal bladder[18]. The mean level of NMP22 in the urine of patients with an active bladder tumor was found to be five times greater than in individuals without bladder cancer[19]. In addition, it may also be elevated in patients with pyuria, urolithiasis, hematuria, cystitis, in the presence of a foreign body, or with bowel segment interposition[3].

While originally designed as a quantitative assay, NMP22 has been developed as a qualitative point-of-care assay. Grossman et al. evaluated NMP22 in comparison to cytology in the detection of bladder cancer in 1331 patients at risk for bladder cancer. This study demonstrated a sensitivity of NMP22 for bladder cancer detection of 55.7% (95% CI, 44.1–66.7%) and a specificity of 85.7% (95% CI, 83–87.6%) compared to 15.8% sensitivity and 99.2% specificity for urinary cytology[20]. Similar results were identified in a subsequent study evaluating NMP22 in the diagnosis of bladder cancer recurrence. In addition, the combination of cystoscopy with NMP22 detected 99.0% of the malignancies[21]. Pooled data from 28 studies and 10,119 patients demonstrated a sensitivity of 68% (95% CI, 62–74%) and a specificity of 79% (95% CI, 74–84%)[7].

As a qualitative point-of-care test, able to provide rapid results within 30–50 min, NMP22 may be useful as an adjunct test to cystoscopy. It has been found to have improved detection of low-grade and non–muscle invasive tumors. The sensitivity for low-grade tumors was higher (83.9%) than for high-grade tumors (62.5%). Sensitivity of NMP22 for non–muscle invasive vs. muscle-invasive disease was also higher (81.8 vs. 57.1%, respectively)[22].

## BLADDER TUMOR ANTIGEN (BTA)

BTA stat and BTA-TRAK are designed to detect bladder tumor–associated antigen in voided urine. This antigen is a human complement factor H–related protein similar to human complement factor H. BTA interacting with complement factor C3b interrupts the complement cascade, potentially conferring a selective growth advantage to cancer cells. In cell culture, normal cells do not express the H-related protein[6]. BTA stat is an immunochromographic, qualitative, point-of-care test similar to NMP22, which is FDA approved for surveillance, but not for screening/diagnosis. BTA-TRAK measures factor H–related protein in a standard, quantitative, ELISA method[3].

The sensitivity and specificity of BTA stat ranged from 67 to 70% and 75 to 78%, respectively. The sensitivity and specificity of BTA-TRAK was noted at 66 and 65%, respectively[23]. BTA stat demonstrates improved sensitivity with increasing histological grade; 53, 76, and 90% for grades 1 through 3. It has also been noted to have improved detection and sensitivity for superficial and low-grade tumors compared to urinary cytology; however, at the cost of lower specificity[6]. As with NMP22, benign conditions of the urinary tract, such as infection or urinary lithiasis, may lead to a false positive for BTA. In addition, false positives may be seen in patients with a history of BCG therapy or urinary diversions utilizing the bowel. After excluding these diseases, the specificity of both BTA stat and NMP22 is significantly increased.

## IMMUNOCYT

Immunocyt utilizes monoclonal antibodies to detect exfoliated urothelial cancer cells in the urine. Three fluorescent-labeled monoclonal antibodies target the M344, LDQ10, and 19A211 antigens, which are specific for bladder carcinoma[24]. The presence of either one red or green fluorescent cell has been commonly used as the criteria indicating a positive result.

Immunocyt has been demonstrated across multiple studies to have high sensitivity and specificity. One prospective study using recurrence as an end point demonstrated a sensitivity of 79.3% for grade 1 tumors, 84.1% for grade 2 tumors, and 92.1% for grade 3 tumors[24]. A systematic review of eight studies across 2896 patients demonstrated an overall sensitivity of 84% (95% CI, 77–91%) and specificity of 75% (95% CI, 78–92%)[7]. When utilized in conjunction with cytology, the sensitivity of Immunocyt was demonstrated to increase from a median of 78 to 88%, with little change in the test specificity. These results have been demonstrated across multiple studies, indicating that Immunocyt is a highly sensitive test that increases the sensitivity of cytology without an appreciable loss of specificity when used together[7,25,26]. These findings have been demonstrated for both low- and high-grade, low-stage tumors.

## LEWIS X

Monoclonal antibodies have also been utilized to detect the Lewis X antigen on exfoliated urothelial cells. The Lewis X is a blood group antigen that is normally absent from urothelial cells in the adult, but is expressed in transitional cell tumors regardless of the secretor status, grade, or stage of the tumor[27]. Commercially available antibodies, such as the P-12 murine monoclonal antibody, have been utilized to target and detect the Lewis X antigen.

A prospective trial evaluating a single voided urine sample from 260 patients demonstrated a sensitivity of 79.8% (95% CI, 70.1–87.4%) and a specificity of 86.4% (95% CI, 80.6–90.9%), detecting 100% of carcinoma *in situ*. In 89 patients, two consecutive urine samples were analyzed, increasing the sensitivity of the Lewis X antigen to 95.1% (95% CI, 84.8–99.2%)[27]. Planz et al. demonstrated similar results from 50 patients with a sensitivity of 84% and a specificity of 80%[28]. A study by Golijanin et al. as well showed that the analysis of two separate urine samples led to an increase of the sensitivity of the Lewis X antigen test from 81.2 to 97% and a specificity of 85.5%[29].

## ACCU-DX

Urinary fibrin/fibrinogen degradation products (FDP) have been noted to be elevated in the presence of bladder malignancy. FDP are produced through the action of plasmin on fibrin and fibrinogen, which leak from the tumor microvasculature. The Accu-Dx test was developed as a qualitative point-of-care immunoassay utilizing murine monoclonal antibodies specific for FDP. However, since these antibodies have also been noted to react with intact fibrinogen typically found in human serum, the usefulness of the test in the presence of hematuria may be low[30].

A multicenter trial evaluating 192 patients with bladder cancer demonstrated a sensitivity of 68.4% and a specificity of 79.6%[31]. A more recent study by Ramakumar et al., involving 196 patients, demonstrated a sensitivity of 52% (95% CI, 38–66%) and a specificity of 91% (95% CI, 85–95%). In this series, the sensitivity of the test for grades 1, 2, and 3 disease was noted to be 25, 46, and 92%, respectively[32].

## TELOMERASE

In recent years, the role of telomerase has been investigated in the pathogenesis of bladder cancer. Telomeres consist of tandem “TTAGGG” repeats at the ends of chromosomes. The gradual loss of these repeats with each cell cycle is known to play an important role in cell death. Telomerase is involved with the synthesis of these tandem repeats and typically inactivated in mature somatic cells. Alteration of this enzyme can result in the immortalization of cell lines in certain malignancies, such as bladder cancer[33]. As such, there has been keen interest in evaluating telomerase as a potential urinary tumor marker.

The enzyme telomerase consists of three subunits: RNA component (hTR), telomerase-associated protein (TP1), and telomerase reverse transcriptase (hTERT). Telomerase has been evaluated as a tumor marker in multiple ways. Telomerase activity has been assessed with a polymerase chain reaction (PCR)–based assay utilizing the telomeric repeat amplification protocol (TRAP). hTR and hTERT assays have also been evaluated as a means of measuring telomerase activity[33].

The TRAP assay has been utilized in multiple studies to evaluate telomerase activity with promising results. In a prospective case control series of 218 men, the sensitivity of telomerase with the TRAP assay was 90% (95% CI, 83–94%) and the specificity was 88% (95% CI, 79–93%). The specificity was increased to 85% for individuals 75 years or younger[34]. A separate study demonstrated similar results with a specificity of 93.5% for the TRAP assay, but a lower sensitivity of 77.4%[35]. For the measurement of the catalytic component of telomerase, hTERT has shown even better results. Sensitivity of this assay has been reported from 84 to 95%, with variable specificity of 65 to 94%[36,37,38,39]. The sensitivity advantage of hTERT over cytology is most pronounced in the detection of low-grade bladder cancer where cytology is known to perform suboptimally[23]. Telomerase appears to be a potentially useful urinary tumor marker; however, there will need to be uniformity and standardization in the assay before it can become useful in clinical practice.

## HYALURONIC ACID (HA)

HA is an extracellular glycosaminoglycan that supports tumor cell adhesion and migration, and offers some protection from immune system surveillance in tumor tissues. Small fragments of HA stimulate angiogenesis and are produced by hyaluronidase (HAase). The small HA fragments have been identified in the urine of patients with bladder cancer[40]. The importance of HA and HAase in bladder cancer pathogenesis has been noted in a recent study in which staining of bladder cancer tissues for HA and HAase correlated with muscle-invasive disease and recurrence[41]. Hyaluronoglucosaminidase-1 (HYAL-1) is a specific HAase that has been identified as a marker for cancer detection, and is a molecular predictor for tumor growth, invasion, and angiogenesis. HYAL-1 mRNA levels have been noted to be elevated 10- to 30-fold higher in bladder cells and tissues that express high HAase activity, as well as in the urine of patients with bladder cancer[42]. As a result, HA and HYAL-1 have been evaluated as potential urinary tumor markers for bladder cancer.

HA and HAase have both been evaluated as possible urinary tumor markers. According to a study by Lokeshwar et al., HA in and of itself has a sensitivity of 83.1%, specificity of 90.1%, and accuracy of 86.5% in detecting bladder cancer regardless of tumor grade. HAase showed a sensitivity of 81.5% and a specificity of 83.8% for higher-grade tumors. Utilizing both HA and HAase tests improved the results in patients regardless of tumor grade. In combination (HA and HAase), the sensitivity was increased to 91.2% for all tumors, with a specificity of 84.4% and an accuracy 88.3%[43]. A follow-up study prospectively evaluated the HA-HAase test compared to BTA stat. This demonstrated similar results for the HA-HAase test as previously reported. In addition, the HA-HAase test was determined to be superior to BTA stat for detecting and predicting tumor recurrence. In a direct comparison, HA-HAase vs. BTA stat, the sensitivity was 94 and 61% and the specificity was 63 and 74%, respectively. A false positive with Ha-HAase was also found to have a significant risk of recurrence within 5 months[40]. When HYAL-1 was specifically evaluated with qualitative PCR, it demonstrated a sensitivity of 92.2% and a specificity of 94.3%. With semi-quantitative PCR, HYAL-1 was not detected in any normal samples and was present in 91.1% of malignant samples with a sensitivity of 91% and a specificity of 100%[44].

## MICROSATELLITE ANALYSIS

Microsatellite analysis evaluates for tumor-specific genomic alterations in a different manner than FISH. Microsatellite analysis targets highly polymorphic, short tandem repeats. This technique evaluates the shift in the normal ratio of two alleles that occurs with genomic alterations from tumor cell transformation. This loss of heterozygosity (LOH) can be used as a biomarker of a suspected neoplastic process. It had already been established that microsatellite changes in urine samples matched DNA extracts from tumor tissues[45].

In a prospective blinded trial, LOH analysis performed significantly better than cytology, particularly for the detection of low-grade tumors. For grade 1 and 2 tumors, the sensitivity of LOH analysis was 60 and 51.8%, respectively, compared to 10 and 18.5% for cytology, respectively. When used in combination with cytology, LOH analysis had an improved sensitivity of 72% for grade 1 and 2 tumors and 96% for grade 3 tumors[45]. A separate study demonstrated very promising results. The overall sensitivity of LOH/microsatellite analysis was 97% compared to 79% for cytology. It was also noted to have significant improvement in the detection of low-grade and -stage bladder tumors, with a sensitivity of 95% for grades G1-G2 and 100% for pTis and pTa tumors[46]. Wild et al. also demonstrated the benefit of combined LOH analysis with voided urine cytology with a sensitivity of 88.2% and a specificity of 97.1%[47].

## BLCA-4

A nuclear matrix protein specific to bladder cancer tissues, termed BLCA-4, has been identified. It holds great promise as a potent urine-based bladder tumor marker. This protein has been found to effect the pathogenesis of bladder cancer by increasing IL-1α (which enhances proliferation and invasion), thrombomodulin (which maintains blood flow for cell survival), and IL-8 (which aids in angiogenesis)[48].

Van Le et al. prospectively evaluated BLCA-4 and determined that it has a sensitivity of 89% and a specificity of 95% for bladder cancer detection using a sandwich-based immunoassay and a predetermined cutoff value[49]. The potential of BLCA-4 as a urinary tumor marker is further strengthened since there is no impact of BLCA-4 levels with a history of urinary tract infection, smoking, catheterization, or cystitis[50].

## PROTEOMICS AND GENOMICS

Advances in the fields of molecular genetics and tissue microarrays have led to the development of many potential novel biomarkers. This has especially been the case within the fields of proteomics and genomics. Proteomics utilizes advanced techniques in high-resolution 2-D electrophoresis, mass spectrometry, and surface-enhanced laser desorption/ionization to identify potential urinary bladder markers that can then be developed into more conventional ELISA tests. The chemokine CXCL-1 and the matrix metalloproteinases MMP-2 and -9 have been identified using these techniques as potential urine-based markers worthy of further study[51].

Genomics is the study of DNA or RNA sequence and gene expression differences resulting in signature expressions for various cancer types. Microarrays have resulted in a shotgun approach that allows a rapid evaluation of multiple DNA or RNA sequences to identify sequences that are potentially characteristic for bladder cancer. Aurora kinase A is an example of a marker identified utilizing these techniques. When evaluated with FISH techniques, this key regulator of mitosis has shown a specificity of 96.6% and a sensitivity of 87% for bladder cancer[51]. Urothelial carcinoma–associated 1 gene has been identified with a sensitivity and specificity of 80.9 and 91.8%, respectively. It is highly sensitive for high-grade noninvasive tumors (91.1%)[50].

## ADDITIONAL URINARY MARKERS

Many other potential biomarkers have been evaluated for their potential usefulness as a urine-based tumor marker. Some of the more promising of these have included cytokeratin CK20, soluble FAS (sFAS), and survivin. The CK20 assay has a 78 to 87% sensitivity and a 56 to 80% specificity for bladder cancer detection, but has poor performance for low-grade tumors. sFAS levels in the urine of patients undergoing surveillance for bladder cancer show higher levels in patients with recurrences and were noted to be more specific than NMP22. Survivin is an antiapoptotic protein that is associated with aggressive cancer behavior. This protein is not expressed in the normal urothelium. Early studies have shown a sensitivity of 64 to 100% and a specificity of 93 to 100%[52]. Human uroplakin 3A (UPK3A) has demonstrated a sensitivity and specificity of 83%[53]. These novel tests demonstrate the potential for further development as novel urinary biomarkers and should be evaluated further to determine their possible clinical utility. A summary of these current and emerging bladder tumor biomarkers, with their respective sensitivities, specificities, and current clinical availability, is shown in Table 1.

**TABLE 1**
**Summary of Urinary Markers and Their Performance Based on Scientific Literature**

| Urinary Marker | Sensitivity (%) | Specificity (%) | Clinical Status | Ref. |
|---|---|---|---|---|
| Cytology | 12.2–79 | 78.4–99.4 | Laboratory | [4,5,6,15,16,20,21,22,24,25,46,47,53] |
| Quanticyt | 42.1–69 | 67.9–87 | Investigational | [8,9,10,11] |
| FISH | 69–92.1 | 89–94.5 | Laboratory | [13,14,15,16] |
| NMP22 | 49.5–92.1 | 66–87.3 | Laboratory and point of care | [16,19,20,21,22,53] |
| BTA-Stat | 50–70 | 67–78 | Point of care | [5,6,14,15,40] |
| Immunocyt | 66.7–84.9 | 62–84.7 | Laboratory | [24,25,26] |
| FDP (Accu-Dx) | 52–68.4 | 79.6–91 | Point of care | [31,32] |
| Telomerase: | | | | |
| TRAP | 77.4–90 | 88–93.5 | Investigational | [34,35] |
| hTERT | 84.8–95 | 43.8–93.5 | | [36,37,38,39] |
| Hyaluronic acid: | | | | |
| HA | 61–83.1 | 53.6–90.1 | Investigational | [41,43] |
| HYAL-1 | 57.6–91 | 78–100 | | [41,44] |
| HAase | 81.5 | 83.8 | | [43] |
| HA/HAase | 88.1–94 | 63–84.4 | | [5,40,43] |
| Lewis X | 79.8–84 | 80–86.4 | Investigational | [27,28,29] |
| Survivin | 75 | 100 | Investigational | [44] |
| LOH | 60–97 | 93 | Investigational | [45,46] |
| BLCA-4 | 89–96.4 | 95–100 | Investigational | [49,50] |
| UPK3A | 83 | 83 | Investigational | [53] |

## CONCLUSION

Bladder cancer continues to be among the top ten malignancies in terms of cancer deaths. Proper diagnosis of this disease is reliant on invasive testing utilizing office cystoscopy and a possible need for biopsy or resection. For patients with a prior history of bladder cancer, surveillance requires routine office cystoscopy to monitor for recurrence. An ideal noninvasive urinary tumor marker would be highly sensitive and specific, minimizing the need for cystoscopy in patients in the absence of bladder cancer. Cytology has been the standard noninvasive urinary tumor marker utilized for decades despite its poor sensitivity, particularly for low-grade tumors. The addition of Urovysion FISH has provided an additional diagnostic armamentarium to determine which patients may need further evaluation. Recently, NMP22 and BTA stat have been developed as point-of-care assays for a more rapid diagnosis to aid both patients and clinicians. Additional assays have been developed that may someday be of sufficient accuracy either as a single, multiple, or a combination of several urinary biomarkers to decrease the need for invasive testing that is necessary to diagnose or detect bladder tumor recurrence. The fields of proteomics and genomics are poised to rapidly identify novel markers that may become part of the clinical armamentarium for clinicians in years to come.
